# The Use of Extracorporeal Organ Support Therapies for Acute Kidney Injury in Cancer Patients

**DOI:** 10.3390/jcm15156024

**Published:** 2026-08-03

**Authors:** Armin Atić, Lui Forni, Vedran Premužić

**Affiliations:** 1 Department of Nephrology, Arterial Hypertension, Dialysis and Transplantation, University Hospital Center Zagreb, 10000 Zagreb, Croatia; 2 Critical Care Unit, Royal Surrey County Hospital, Guildford GU2 7XX, UK; 3 School of Medicine, University of Surrey, Guildford GU2 7XH, UK; 4 School of Medicine, Catholic University of Croatia, 10000 Zagreb, Croatia

**Keywords:** acute kidney injury, neoplasms, renal replacement therapy, blood purification, critical illness

## Abstract

Acute kidney injury (AKI) is a frequent and serious complication in patients with cancer, associated not only with increased morbidity and mortality, but also with the interruption or modification of anticancer therapy. In critically ill patients with cancer, AKI arises from a complex interplay of cancer-related, treatment-related, and patient-related factors, with many patients requiring renal replacement therapy (RRT) and admission to the intensive care unit (ICU). This narrative review summarizes the etiology of AKI in patients with cancer and discusses the role of extracorporeal organ support (ECOS) therapies, with particular emphasis on continuous renal replacement therapy (CRRT). Understanding the underlying etiology of AKI in this patient population is essential for guiding treatment decisions, particularly when therapeutic plasma exchange, hemadsorption, or other extracorporeal modalities are considered which may directly target the pathophysiology driving organ dysfunction. Although observational data suggest that ECOS use may improve both short- and long-term outcomes, robust evidence demonstrating consistent benefit remains lacking. Furthermore, limited pharmacokinetic data for anticancer drugs in patients undergoing dialysis or RRT creates significant uncertainty regarding optimal dosing strategies. By providing a structured overview of extracorporeal organ support strategies in cancer-associated AKI, this review aims to support multidisciplinary decision-making and facilitate more individualized critical care for this high-risk population.

## 1. Introduction

Acute kidney injury (AKI) is a common and clinically significant complication in patients with cancer, associated with increased morbidity, mortality, prolonged hospitalization, and disruption or cessation of cancer-directed therapy. The incidence of AKI is particularly high in critically ill patients with cancer, reflecting the complex interaction between advancing age, comorbidities, disease-related factors—including cancer type, treatment stage, and prognosis—and acute systemic insults such as sepsis, treatment-related toxicity, and multiorgan failure. Consequently, a substantial proportion of patients with cancer admitted to the ICU will require RRT during their stay.

The management of AKI in patients with malignancy is uniquely challenging. Prognosis is determined not only by the severity and potential reversibility of kidney injury, but also by the cancer type and stage, the goals of ongoing cancer therapy, baseline functional status, and the likelihood of meaningful clinical recovery. Decisions regarding escalation of care—including ICU admission and initiation of extracorporeal organ support—should be multidisciplinary, involving renal, oncological, and critical care physicians, whilst remaining aligned with patient preferences and overall aims of care, so as to avoid disproportionately burdensome treatment.

This narrative review summarises the common etiologies of AKI in patients with hematological and solid malignancies, with a focus on the management of these patients using extracorporeal organ support therapies. Particular emphasis is placed on the pathophysiological rationale underpinning therapeutic choices, the need for individualised and multidisciplinary decision-making, and the limitations of the current evidence base.

## 2. Etiology of Acute Kidney Injury in Cancer Patients

Defining the etiology of AKI in any patient is essential and patients with cancer are no exception, as it has direct implications for the selection of ECOS modality and for prognosis. AKI in this context may arise from the malignancy itself, from complications such as infection, treatment-related organ toxicity, or systemic dysfunction affecting the heart, liver, or other organs.

### 2.1. Volume Imbalance

Many patients with cancer present with hypovolemia arising from reduced oral intake, gastrointestinal losses, or relative intravascular volume depletion secondary to sepsis, cardiac failure, side effects of treatment or capillary leak syndrome—all of which are prevalent in this population [1]. Cardiotoxic effects of certain chemotherapeutic agents may precipitate acute or chronic cardiac dysfunction, resulting in cardiorenal syndrome. Hepatic involvement from metastatic disease, sinusoidal obstruction syndrome following hematopoietic stem cell transplantation (HSCT), or treatment-induced liver injury may give rise to the pathophysiology of hepatorenal syndrome and secondary AKI [2]. These functional forms of kidney injury may be reversible initially but can progress to intrinsic renal damage if not recognised and treated promptly. Hypercalcaemia, a common presentation in many cancer patients, may lead to afferent arteriolar constriction, thereby producing functional prerenal AKI.

### 2.2. Intrisic Renal Disease

**Figure 1:**
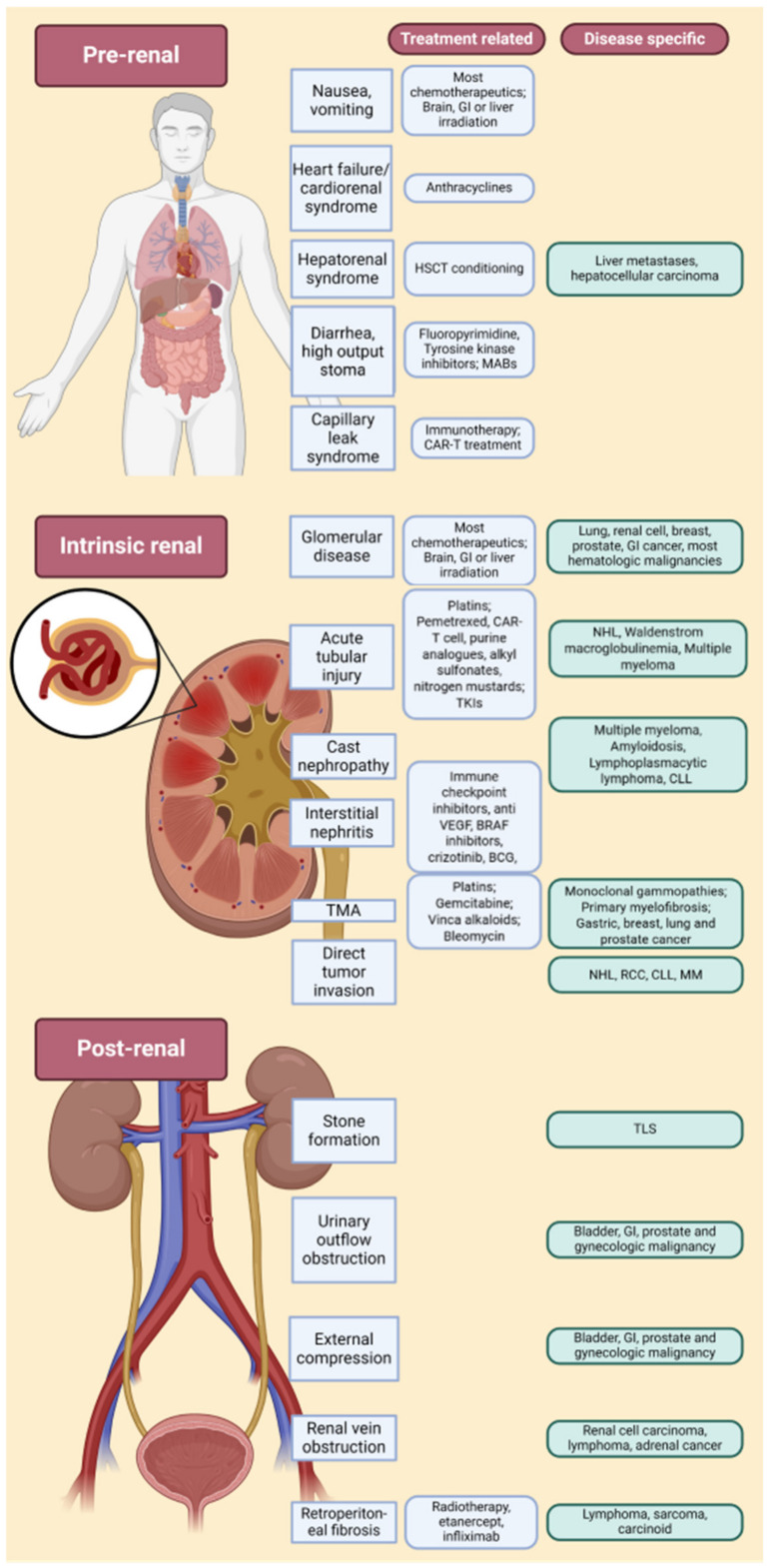
Causes of acute kidney injury in patients with cancer. HSCT—Hematopoietic Stem Cell Transplantation, CAR-T—Chimeric Antigen Receptor—T cell, GI—Gastrointestinal, MAB—monoclonal antibodies, NHL—Non-Hodgkin Lymphoma, MM—Multiple Myeloma, CLL—Chronic Lymphocytic Leukaemia, VEGF—Vascular Endothelial Growth Factor, BCG—Bacillus Calmette–Guerin, TMA—Thrombotic Microangiopathy, RCC—Renal Cell Carcinoma, TLS—Tumor Lysis Syndrome.

A wide spectrum of intrinsic renal diseases, encompassing both glomerular and tubulointerstitial pathology, may occur in patients with malignant disease. Acute tubular necrosis (ATN) can arise as a consequence of disease-related complications (such as sepsis or hypovolemia) or from direct tubular injury caused by cancer-specific or other therapies (Figure 1).

Glomerular diseases frequently develop secondary to malignancy, and in some cases, the cancer is identified during the investigation of a patient presenting with features of glomerulopathy. An important consideration in paraneoplastic glomerular disease is its relationship with the underlying malignancy: effective treatment of the cancer may result in meaningful improvement of the glomerulopathy. Glomerular disease may occur in both solid and hematological malignancies, and may also arise as a consequence of anticancer treatment. Certain cancer types are more commonly associated with specific glomerular diseases and vice versa; however, a variety of paraneoplastic glomerular lesions may occur with any cancer type (Supplementary Table S1) [3,4,5,6]. Direct intraglomerular metastasis is rare, occurring most frequently with lung cancer [3,7]. In hematological malignancies, deposition diseases, membranoproliferative glomerulonephritis, minimal change disease (MCD), and membranous nephropathy (MN) are among the most frequently encountered lesions, whilst diseases such as lymphoma may also cause direct renal parenchymal infiltration. Nephrotic syndrome occurs in 0.4–6.0% of patients treated with HSCT, most commonly in association with established graft-versus-host disease (GVHD), with membranous nephropathy being the most common glomerular lesion in this setting [4].

Several of these mechanisms may coexist and contribute to multifactorial AKI. Hypercalcaemia, beyond its prerenal effects, can also induce hypertension or exacerbate tubular injury. Tumor lysis syndrome (TLS), a hematological emergency, results from the massive release of tumor cell contents into the circulation. Characteristic biochemical features include hyperuricemia, hypocalcemia, hyperphosphatemia, and hyperkalemia. AKI occurs in approximately two-thirds of patients with TLS and is associated with worse outcomes [8]. Multiple mechanisms underlie TLS-associated AKI, including crystal deposition (calcium phosphate, uric acid, and xanthine), although the widespread adoption of rasburicase in at-risk patients has made crystal-mediated AKI less common. Emerging evidence suggests that extracellular histones and endothelial dysfunction also play important roles in TLS-associated renal injury [9].

Capillary leak syndrome (CLS) is characterised by increased capillary permeability leading to the redistribution of protein-rich fluid from the intravascular compartment, driven by widespread cytokine release. CLS is increasingly encountered in the context of expanding use of monoclonal antibodies and gemcitabine. The resulting intravascular volume depletion activates the renin–angiotensin system and causes effective prerenal AKI; cytokine-mediated tubular injury may simultaneously result in ATN, while the development of large ascites may worsen renal function through abdominal compartment syndrome [10].

Cancer therapy may itself directly cause AKI through diverse mechanisms, including acute interstitial nephritis, drug-induced lupus, ATN, and glomerular disease. Glomerular disease attributable to chemotherapy is summarised in Supplementary Table S1. Other pharmacological agents commonly used in cancer patients for prophylaxis or supportive care—such as non-steroidal anti-inflammatory drugs (NSAIDs), proton pump inhibitors, antibiotics, and antiviral agents—may also contribute to nephrotoxicity [11,12]. Postrenal causes of AKI in patients with malignancy typically arise from urinary tract obstruction due to calculi, extrinsic compression, or renal vein occlusion. Surgical management of the obstructing pathology usually results in resolution of AKI in the majority of cases.

## 3. Admission to the Intensive Care Unit

The threshold for ICU admission of patients with malignant disease has changed considerably in recent years, underpinned by evidence demonstrating comparable ICU mortality between patients with cancer and those with other serious chronic conditions, such as heart failure and liver cirrhosis [13,14]. Even the use of advanced life-sustaining therapies such as extracorporeal membrane oxygenation (ECMO) has been shown to offer favourable short- and long-term outcomes in selected patients with hematological malignancies [15]. Improvements in overall cancer treatment outcomes, advances in ICU management, and evidence supporting earlier admission for multiorgan failure have collectively shifted the paradigm surrounding ICU care for patients with cancer [13,15]. Nevertheless, data on long-term quality of life following ICU admission, and the effect of ICU care on disease remission or disease-free survival, remain limited. Significant challenges persist, and careful patient selection, clear and compassionate communication, and an open interdisciplinary approach remain paramount when determining the appropriateness and level of ICU care.

## 4. Renal Replacement Therapy in Patients with Acute Kidney Injury and Cancer

The expanding therapeutic landscape for cancer has led to a growing number of patients with malignant disease being admitted to ICUs. Approximately one in every twenty patients with cancer will develop critical illness requiring ICU admission [16]. AKI is highly prevalent in this population, with a 27% five-year risk demonstrated in a large Danish cohort; of those who develop kidney injury, approximately 5% will require CRRT [17]. Current estimates suggest that up to 40% of critically ill patients with cancer may require some form of RRT [1,17,18]. Importantly, the need for RRT in cancer patients admitted to the ICU is associated with substantially worse survival [19,20].

The optimal choice of RRT modality and the timing of its initiation remain subjects of considerable debate across all patient populations, not least in those with cancer. In this setting, however, disease prognosis, ongoing specific anticancer treatment, and future therapeutic options introduce additional complexity, necessitating involvement of hematology and oncology specialists in the decision-making process. Furthermore, advances in hemadsorption and extracorporeal blood purification (EBP) techniques have introduced novel therapeutic approaches that may improve clinical outcomes in selected patients [21]. Hemadsorption refers to the passage of blood, or of an ultrafiltrate, through a cartridge containing a porous sorbent that binds target solutes—such as cytokines, bilirubin, myoglobin or certain drugs—by hydrophobic and size-selective interactions, whereas extracorporeal blood purification (EBP) is a broader term encompassing hemadsorption together with other sorbent- and membrane-based techniques used to remove circulating mediators of organ injury.

### 4.1. Indications for Renal Replacement Therapy

The classical indications for RRT—refractory metabolic acidosis, hyperkalaemia, volume overload, uraemic symptoms, and intoxication with dialysable agents—are generally not disputed in the critical care community and, in the absence of contraindications, should prompt initiation of treatment. However, RRT is frequently commenced in the absence of these criteria based on clinical judgement. Relative indications include organ dysfunction attributable to hypervolemia (e.g., respiratory failure), anticipated solute burden (e.g., rhabdomyolysis), severe underlying disease, poor tolerance of AKI consequences, the need for large volume fluid administration or pharmacological agents, and accumulation of toxins or drugs amenable to extracorporeal clearance [21]. In cancer patients, specific complications related to the malignancy or its treatment may create additional indications for RRT initiation; these predominantly influence the choice of modality rather than the decision to treat. In particular, patients receiving chimeric antigen receptor (CAR) T-cell therapy may develop grade 3–4 cytokine release syndrome (CRS) that is refractory to tocilizumab and corticosteroids, driven by a massive systemic inflammatory response. In such cases, continuous renal replacement therapy combined with extracorporeal cytokine adsorption has been used in an attempt to remove inflammatory mediators such as IL-1 and IL-6 [22].

### 4.2. Timing

The goals of RRT in critically ill patients with cancer include optimisation of fluid balance, electrolyte and acid–base homeostasis, and removal of uraemic and other toxins. Although earlier initiation of RRT (before the onset of classical indications) may confer potential benefits, it is not without risk: additional vascular access, exposure to extracorporeal circulation, and anticoagulation all carry associated hazards [23]. Furthermore, in patients with a very low likelihood of survival, RRT may paradoxically contribute to worse outcomes [24,25].

The absence of large randomised controlled trials using uniform definitions and endpoints has hampered the development of clear recommendations for RRT timing. Significant progress has been made through landmark trials including STARRT-AKI, ELAIN, and AKIKI, which have demonstrated the feasibility of such investigations [26,27,28]. Two of these trials (AKIKI and STARRT-AKI) found no mortality benefit with earlier RRT initiation, whereas the ELAIN trial reported a benefit associated with an earlier start. Direct comparison between these trials is limited by differences in the definitions of early versus delayed RRT and in inclusion criteria, resulting in treatment approaches that may not reflect routine clinical practice [24]. These studies collectively highlight the importance of integrating the overall clinical context—including AKI aetiology—into the decision to initiate RRT. Specific conditions such as rhabdomyolysis or TLS, or defined courses of drug toxicity, may prompt earlier initiation and also allow for RRT discontinuation at clearly defined timepoints once the precipitating factor has been adequately addressed.

### 4.3. Selecting the RRT Modality

**Figure 2:**
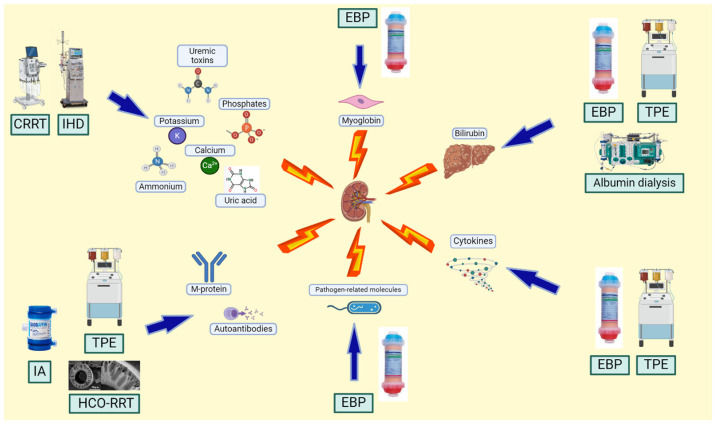
The role of different organ support modalities in the removal of specific target molecules. CRRT—Continuous Renal Replacement Therapy, IHD—Intermittent Haemodialysis, EBP—Extracorporeal Blood Purification, TPE—Therapeutic Plasma Exchange, IA—Immunoadsorption, HCO-RRT—High Cut-Off Renal Replacement Therapy, M-protein—Monoclonal protein.

RRT encompasses numerous renal and non-renal indications in patients with malignant disease, and familiarity with available modalities and their indications is essential for optimal management. The decision to use continuous renal replacement therapy (CRRT) rather than intermittent renal replacement therapy (IRRT) is typically driven by hemodynamic instability [23]. CRRT can be delivered as continuous veno-venous hemofiltration (CVVH), hemodialysis (CVVHD) or hemodiafiltration (CVVHDF), and the choice is guided by the clinical goal: convective modalities such as CVVH are favoured in shock and fluid overload, whereas CVVHD or CVVHDF, which add a diffusive component, are preferred when solute clearance is the priority, for example in tumour lysis syndrome, in cisplatin-associated AKI, or when methotrexate must be removed in a hemodynamically unstable patient. The choice of CRRT modality must account for the target molecule to be removed (Figure 2), and the presence of additional organ failure (e.g., hepatic failure) or paraneoplastic syndromes may necessitate the use of hemadsorption, albumin dialysis, or plasma exchange alongside RRT.

There remains a significant knowledge gap regarding the dosing and dialysability of chemotherapeutic agents. This gap reflects, in part, the historical exclusion of patients with chronic kidney disease or end-stage renal disease from clinical trials during drug development, and the paucity of direct pharmacokinetic studies examining drug concentrations in the context of renal failure and extracorporeal therapy [29].

Chemotherapy may be continued in the ICU in patients whose critical illness is driven by their underlying malignancy, or in those for whom scheduled or essential treatment cannot be deferred [21]. In such circumstances, knowledge of the dialysability and pharmacokinetics of the relevant agents is essential to avoid either underdosing or toxicity. Data on chemotherapy dosing in dialysis patients are sparse; many agents undergo renal excretion and require dose adjustment, yet evidence to guide prescribing in the dialysis setting is often unavailable. A multicentre study (CANDY—CANcer and DialYsis) found that 72% of patients received a chemotherapeutic agent requiring dose modification for which no dialysis-specific data existed, and 44% of all patients experienced toxicity attributed to chemotherapy [30]. The complexity is further compounded by the need to administer certain agents after dialysis, which may complicate scheduling in patients receiving continuous therapies.

For some of the most commonly used agents, dosing recommendations for end-stage renal disease are available, providing a basis for safer prescribing [31]. With respect to CRRT specifically, no studies have formally evaluated appropriate dosing in relation to the CRRT modality employed. Nonetheless, data derived from intermittent dialysis patients offers partial guidance and can inform clinical decision-making. For drugs that are effectively cleared by extracorporeal therapy—such as methotrexate or cyclophosphamide—and are required for adequate treatment of the underlying cancer, IRRT-based strategies may be preferable where feasible. When chemotherapy toxicity itself necessitates extracorporeal treatment, CRRT or other blood purification techniques may be employed, using a systematic approach analogous to that used in poisoning management [32].

## 5. Extracorporeal Organ Support Therapies in Patients with Cancer

Outcomes for critically ill patients with various malignant diseases continue to improve [33]. ICU admission in this population frequently involves multiple modes of organ support in addition to RRT, including mechanical ventilation (MV), extracorporeal membrane oxygenation (ECMO), and cardiovascular support. Beyond these critical care interventions, a number of other extracorporeal treatment options have an established or emerging role in the management of cancer patients.

Complications arising from antibody or immune complex formation may be treated with total plasma exchange (TPE) or immunoadsorption (IA). TPE is also the first-line intervention for symptomatic hyperviscosity syndrome, most often encountered in Waldenström macroglobulinemia, where it rapidly reduces circulating IgM and reverses the clinical manifestations pending disease-directed therapy [34]. Thrombotic microangiopathy (TMA) in cancer patients may occur as a consequence of the malignancy itself or of its treatment—including pharmacological therapies and bone marrow transplantation. Several distinct mechanisms and injury patterns have been described in cancer drug-associated TMA [35]. Classic chemotherapeutic agents typically cause dose-dependent toxicity, presenting with the typical clinical syndrome of TMA accompanied by high rates of renal failure. Newer agents such as anti-VEGF therapies appear to cause more prominent endothelial injury, with a different phenotype characterized by hypertension and proteinuria in the absence of a significant decline in renal function [36]. Management involves removal of the causative agent where applicable, pharmacological treatment (such as rituximab or complement inhibitors), and interventions targeting the removal of immune complexes, including therapeutic plasma exchange, immunoadsorption, or in some cases haemodialysis and exchange transfusion. TMA is associated with high rates of renal failure, which itself confers a worse prognosis [36,37,38]. The mainstay of management depends on the trigger: malignancy-induced TMA is treated primarily by initiating effective chemotherapy against the underlying cancer, whereas chemotherapy- or immunotherapy-induced TMA is managed principally by discontinuing the offending agent. Despite their common use, the efficacy of plasma exchange and immunoadsorption in TMA is modest (approximately 36% and 45% respectively), and targeted pharmacological therapy may be required [37]. The evidence base for determining the optimal timing and duration of extracorporeal therapy in this context remains limited; however, the absence of a clinical response should prompt consideration of treatment discontinuation.

AKI occurs in 15–40% of patients with multiple myeloma (MM), and renal recovery is associated with improved survival [39]. Studies have demonstrated that renal recovery in MM is dependent on achieving an early reduction in serum free light chain (FLC) concentrations, which can be accomplished by effective chemotherapy and through either plasma exchange or the use of high-cutoff hemodialysis membranes [40,41]. Notably, randomised data have not shown a clear benefit of routine plasma exchange in myeloma cast nephropathy, and high cut-off haemodialysis (HCO-HD) has emerged as an alternative extracorporeal approach owing to its greater capacity to clear serum free light chains [42]. Either approach, when combined with bortezomib-based therapy, facilitates renal recovery commensurate with the degree of FLC reduction achieved [41]. In patients receiving bortezomib-based therapies, the addition of HCO versus standard high-flux hemodialysis did not improve kidney recovery [42]. In the absence of studies directly comparing these two modalities, the relative contribution of TPE to renal and patient outcomes remains uncertain.

## 6. The Use of Hemadsorption Techniques in Cancer-Associated AKI

As hemadsorption technologies have advanced, the range of potential indications for extracorporeal blood purification (EBP) has broadened. EBP is now used or under investigation for the treatment of sepsis, rhabdomyolysis, liver failure, drug toxicity, and the removal of cytokines and endotoxins, among other applications [43,44,45,46,47,48,49,50,51]. These clinical scenarios occur frequently in patients with cancer, and appropriately selected EBP may offer therapeutic benefit across several domains.

Infections, superinfections, and sepsis occur at higher rates in cancer patients than in the general population [52,53]. EBP can enable non-selective cytokine removal and pathogen binding [54,55]. Bacterial endotoxins, damage-associated molecular patterns (DAMPs), and other drivers of inflammatory dysregulation represent additional targets for EBP therapy. As described above, AKI in haemato-oncological patients may arise from multiple aetiologies, many of which share cytokine-mediated pathways as a common mechanism. Selective cytopheretic devices (SCD) offer targeted sequestration of activated circulating monocytes, thereby modulating the immune response and attenuating organ damage driven by excessive immune activation [56]; preliminary evidence supporting this approach has emerged from studies in paediatric AKI [57].

Rhabdomyolysis in cancer patients typically occurs as a consequence of cytotoxic drug toxicity or paraneoplastic myositis; the use of certain haemadsorbers—such as CytoSorb—has shown promising results in terms of renal recovery in patients with severe rhabdomyolysis [58,59,60]. Hepatic failure is another recognised complication in cancer patients, arising from drug toxicity, direct tumour involvement, or Budd–Chiari syndrome, or as a component of multiorgan failure [61]. To date, the most extensively studied extracorporeal approach for acute-on-chronic liver failure is plasma exchange, which acts principally through the elimination of pro-inflammatory cytokines; albumin dialysis has demonstrated some benefit in patients with liver failure and concurrent AKI [62,63]. In this context, EBP offers several potential advantages, including removal of accumulated toxic substances such as bile acids and attenuation of the immunological cascade underpinning liver failure [46]. Although uncertainties regarding patient selection and optimal EBP protocols remain, carefully selected patients with cancer may derive benefit across multiple domains—including toxin removal, immune modulation, and pathogen clearance.

## 7. Conclusions

AKI is a frequent and prognostically important complication in patients with cancer, arising from a complex interplay of malignancy-related, treatment-related and patient-related factors. Because the underlying etiology directly determines both prognosis and the most appropriate form of organ support, a careful, individualised diagnostic approach is essential. Continuous renal replacement therapy remains the cornerstone of extracorporeal support in the critically ill patient with cancer, while additional modalities—such as therapeutic plasma exchange, high cut-off haemodialysis, hemadsorption and other blood purification techniques—may be selected to target specific pathophysiological processes. Although observational data suggest that these therapies can improve outcomes in selected patients, high-quality randomised evidence is still lacking, and important uncertainties persist regarding patient selection, optimal timing, and the dosing and dialysability of anticancer agents. Decisions to initiate or escalate organ support should therefore remain multidisciplinary—integrating nephrology, oncology/haematology and critical care expertise—and firmly aligned with the goals of care of each patient. Well-designed prospective studies are needed to define which patients derive genuine benefit from extracorporeal organ support and to translate these techniques into evidence-based practice.

## Data Availability

No new data were created or analyzed in this study.

## Supplementary Materials

The following supporting information can be downloaded at https://www.mdpi.com/article/10.3390/jcm15156024/s1. Supplementary Table S1: Intrinsic renal disease associated with haemato-oncological diseases or their treatment.

## Abbreviations

The following abbreviations are used in this manuscript:

AKI	Acute Kidney Injury
ATN	Acute Tubular Necrosis
BCG	Bacillus Calmette–Guerin
CAR-T	Chimeric Antigen Receptor—T cell
CLL	Chronic Lymphocytic Leukaemia
CLS	Capillary Leak Syndrome
CRRT	Continuous Renal Replacement Therapy
CRS	Cytokine Release Syndrome
CVVH	Continuous Veno-Venous Hemofiltration
CVVHD	Continuous Veno-Venous Hemodialysis
CVVHDF	Continuous Veno-Venous Hemodiafiltration
EBP	Extracorporeal Blood Purification
ECMO	Extracorporeal Membrane Oxygenation
ECOS	Extracorporeal Organ Support
FLC	Free Light Chain
FSGS	Focal Segmental Glomerulosclerosis
GVHD	Graft-versus-Host Disease
HCO-HD	High Cut-Off Hemodialysis
HF-HD	High-Flux Hemodialysis
HSCT	Hematopoietic Stem Cell Transplantation
IA	Immunoadsorption
ICU	Intensive Care Unit
IRRT	Intermittent Renal Replacement Therapy
MAB	Monoclonal Antibodies
MCD	Minimal Change Disease
MM	Multiple Myeloma
MN	Membranous Glomerulonephritis
MPGN	Membranoproliferative Glomerulonephritis
MV	Mechanical Ventilation
NHL	Non-Hodgkin Lymphoma
NSAID	Non-Steroidal Anti-Inflammatory Drug
PD-1	Programmed cell Death protein 1
RCC	Renal Cell Carcinoma
RPGN	Rapidly Progressive Glomerulonephritis
RRT	Renal Replacement Therapy
SCD	Selective Cytophoretic Device
TKI	Tyrosine Kinase Inhibitors
TLS	Tumor Lysis Syndrome
TMA	Thrombotic Microangiopathy
TPE	Total Plasma Exchange
VEGF	Vascular Endothelial Growth Factor
WM	Waldenström Macroglobulinemia
