# Supplementary material for: The Use of Extracorporeal Organ Support Therapies for Acute Kidney Injury in Cancer Patients
Source: J Clin Med. 2026 Aug 3;15(15):6024. doi: 10.3390/jcm15156024 (PMC13466599; doi:10.3390/jcm15156024)
Supplement: Supplementary file 1 [file jcm-15-06024-s001.zip › jcm-4439975-supplementary.pdf]

Supplementary Table S1. Intrinsic renal disease associated with haemato-oncological diseases or their treatment.

Glomerular Disease	Associated Haemato-Oncologic Disease	Associated Cancer Treatment
Membranous nephropathy	Lung cancer, GI cancers, Breast cancer, Prostate, CLL, GVHD, NHL	Monoclonal antibodies
Minimal change disease	Hodgkin lymphoma, NHL, Thymoma, CLL	Etanercept, gold, anti-VEGF, PD-1 inhibitors (nivolumab, pembrolizumab)
Focal segmental glomerulosclerosis	GVHD, Hodgkin lymphoma, NHL	Anti-angiogenesis drugs, Interferon
Membranoproliferative glomerulonephritis	RCC, Lung cancer, CLL, NHL, WM	Gemcitabine, interferon-α/β, pembrolizumab
IgA nephropathy/vasculitis	Lung cancer, Head and neck cancers	Immune checkpoint inhibitors, anti-TNF-α agents
Rapidly proliferative glomerulonephritis	RCC, lung cancer, thymoma	Immune checkpoint inhibitors
Thrombotic microangiopathy	Gastric, breast, prostate, lung, pancreas, Tumour invasion of bone marrow, POEMS syndrome, monoclonal gammopathy, primary myelofibrosis, CLL	Tyrosine kinase inhibitors, Platinums, bleomycin, 5-FU, Vincristine, proteasome inhibitors, anti-VEGF, mitomycin C, anti-angiogenesis agents
Immunotactoid glomerulopathy	NHL, CLL, MM	
Fibrillary glomerulonephritis	NHL, Polyclonal haematologic malignancies	
Pauci-immune vasculitis		Immune checkpoint inhibitors

GI—Gastrointestinal, NHL—Non-Hodgkin Lymphoma, WM—Waldenström Macroglobulinemia, MM—Multiple Myeloma, CLL—Chronic Lymphocytic Leukaemia, VEGF—Vascular Endothelial Growth Factor, RCC—Renal Cell Carcinoma, GVHD—Graft versus host disease, 5-FU—5—fluorouracil, POEMS—Polyneuropathy, Organomegaly, Endocrinopathy, Monoclonal protein, and Skin changes.
